# Extracellular Vesicle-Derived Bioactive Molecules for Corneal and Ocular Surface Regeneration

**DOI:** 10.3390/jcm14155594

**Published:** 2025-08-07

**Authors:** Ana Kolenc, Živa Dimnik, Miha Marzidovšek, Petra Schollmayer, Marko Hawlina, Elvira Maličev, Zala Lužnik Marzidovšek

**Affiliations:** 1 Blood Transfusion Centre of Slovenia, 1000 Ljubljana, Slovenia; ana_kolenc@ztm.si (A.K.); ziva.dimnik@ztm.si (Ž.D.); elvira.malicev@ztm.si (E.M.); 2 Medical Faculty, University of Ljubljana, 1000 Ljubljana, Slovenia; marko.hawlina@gmail.com; 3 Eye Hospital, University Medical Centre Ljubljana, 1000 Ljubljana, Slovenia; miha.marzidovsek@kclj.si (M.M.); petra.schollmayer@gmail.com (P.S.); 4 Biotechnical Faculty, University of Ljubljana, 1000 Ljubljana, Slovenia

**Keywords:** extracellular vesicles, bioactive molecules, cornea, dry eye disease, miRNA, mesenchymal stem cells, ophthalmology

## Abstract

Cell-based therapies emerge as potential treatment options for various debilitating diseases. Preclinical research and clinical studies involving cells increased exponentially in the past decade. In addition to cell-based approaches, the use of extracellular vesicles (EVs), which are released by nearly all cell types, emerged as a promising cell-free alternative. Those approaches are also being explored in the field of ophthalmology. Several clinical trials involving EVs are underway to develop potential treatments for advanced ocular surface diseases, including corneal disorders, injuries, and dry eye disease. The cargo carried by EVs has been shown to include a diverse array of functional molecules such as transcription factors, cytokines, growth factors, mRNA, tRNA, rRNA, miRNA, and fragments of dsDNA. While the molecular composition of EVs is already well characterised, the specific activity of these molecules upon delivery to recipient cells remains poorly understood. In this review, we summarise recent studies investigating the bioactive molecules within EVs shown to influence or modulate cellular activity on the ocular surface. Among these, various miRNAs have most commonly been identified as therapeutic agents targeting distinct molecular pathways. The EVs studied were predominantly derived from various mesenchymal stem cells.

## 1. Introduction

The cornea, an avascular and immune-privileged structure, is the outermost transparent tissue of the eye, which plays a crucial role in light transmission and visual clarity. Due to its direct contact with the external environment, it is constantly amenable to injuries, and thus requires an inherent ability to repair tissue damage. Structurally, the cornea consists of five layers: the squamous epithelium, Bowman’s membrane, stroma, Descemet’s membrane, and the innermost corneal endothelium [1].

The outer corneal epithelial layer is renewed by a small population of self-renewing stem cells (SCs) primarily found at the densely innervated peripheral corneal area called the limbus [2,3]. Corneal nerves help maintain the integrity of corneal surface by releasing epitheliotropic substances that promote corneal SC survival and epithelial regeneration [3]. On the other hand, corneal endothelium, derived from neural crest cells, has very low proliferative potential in vivo [1]. Substantial loss or dysfunction of endothelial cells leads to pathological corneal edema, leading to visual impairment [1]. Thus, corneal repair is a complex process involving multi-layered regulatory scenarios and cell types [4].

Severe corneal damage can occur as a consequence of several clinical conditions, such as trauma or chemical injuries, infections, systemic diseases, age-related degenerations, and corneal dystrophies. All these factors, along with inefficient tissue repair processes trigger corneal scarring and neovascularisation that can lead to complete loss of vision, compromising patient’s quality of life and putting an immediate burden on the healthcare systems [4,5]. Corneal diseases are the fifth leading cause of blindness globally, affecting approximately 4.5 million people [5].

In case of treatable corneal blindness, corneal transplantation using donor tissue is the standard intervention, with about 180,000 procedures performed annually [6]. However, challenges remain due to donor tissue shortages, storage limitations, and the limited longevity of grafted endothelial cells [6]. Moreover, in cases of limbal stem cell deficiency, transplantation fails to restore the corneal surface in the long term since corneal grafts lack regenerative stem cells. This highlights the critical need for new therapeutic approaches to address corneal blindness and improve outcomes for patients suffering from severe ocular surface and corneal disorders.

Over the past decade, stem cell-based therapies emerged as transformative tools in various medical fields including ophthalmology. Stem cell-based therapies have been tested for advanced ocular surface diseases such as severe dry eye syndrome, corneal graft rejection, limbal stem cell deficiency, and graft-versus-host disease [7]. Stem cells, particularly mesenchymal stem cells (MSCs), show promise due to their regenerative, immunomodulatory, and paracrine properties, which can influence damaged tissue regeneration on several levels. They can be used as an extra-ocular SC source to directly replace the damaged corneal cells or can function in a paracrine way by stimulating endogenous tissue regeneration capacity, mediating the effect through direct cell–cell contact or releasing bioactive molecules into the environment.

MSCs both in vivo and during cultivation in cell culture release chemokines, cytokines, growth factors, and immunomodulatory proteins, such as interleukin-6 (IL-6), hepatocyte growth factor (HGF), prostaglandin E2 (PGE2), transforming growth factor β (TGF-β), programmed death ligand 1 (PD-L1), indoleamine 2,3-dioxygenase (IDO), tumour necrosis factor-α-stimulated gene 6 (TSG-6), interleukin-10 (IL-10), tumour necrosis factor α (TNF-α), matrix metalloproteinase-9 (MMP-9), and soluble human leukocyte antigen-G isoform 5 (sHLA-G5) [8]. Most of these molecules are packaged and released in extracellular vesicles (EVs), safely delivering biomolecules to target cells.

Thus, EVs emerged as promising candidates for novel and safer cell-free alternative therapeutic approaches to MSC administration in ophthalmology, overcoming some safety concerns such as severe immune reactions or ectopic tissue formation. Preclinical evidence indicates that these vesicles can modulate inflammation, promote tissue repair, and support nerve regeneration, highlighting their potential in treating various ocular surface disorders, including corneal injuries and dry eye syndrome. Specifically in the context of corneal healing, EVs demonstrated the ability to reduce apoptosis, inflammation, and corneal angiogenesis, while simultaneously enhancing corneal epithelial cell proliferation, survival, extracellular matrix synthesis, and the migration and differentiation of limbal stem cells [9].

This review aims to provide a comprehensive overview of EVs’ molecular composition and biological functions of specific molecules that have been studied so far, with a particular focus on EV application in treating corneal wound healing and ocular surface diseases.

## 2. Extracellular Vesicles

EVs are lipid bilayer-enclosed, nano- to microscale particles released by all cell types under physiological and pathological conditions [10]. They carry diverse biomolecules, including lipids, proteins, mRNAs, miRNAs, circRNAs, and other non-coding RNAs [11]. EVs are classified into several subtypes based on their biogenesis and size: microvesicles (100–1000 nm), which bud directly from the plasma membrane; apoptotic bodies (500–5000 nm), formed during programmed cell death; and exosomes (under 200 nm), which originate from endosomal compartments [12].

EVs garnered particular interest due to their role in mediating intercellular communication via paracrine signalling. Their properties—including low immunogenicity, minimal risk of tumourigenicity, prolonged circulation half-life, and the ability to traverse the blood–brain barrier—underscore their promise as both diagnostic and therapeutic tools in clinical applications, circumventing many ethical issues associated with cell therapies. Moreover, genetic engineering could modify the cells, and new therapeutic factors could be introduced into their EVs [12]. EVs can be isolated and stored long-term at low temperatures, eliminating the need to produce large amounts of cells during inoculation, which is required for cellular therapy [13]. Throughout this manuscript, we refer to all vesicular preparations as EVs, as the specific terms listed above are often used without direct experimental confirmation of their origin, and distinguishing between these partially size-overlapping subclasses remains technically challenging [10].

EVs derived from various stem and progenitor cell populations demonstrated promising results as cell-free therapies in preclinical models. Stem cell-derived EVs have been shown to promote angiogenesis in ischemic conditions, tissue repair, neuroprotection, and to regulate immune responses [14,15,16,17]. Indeed, several mechanisms of action have been proposed for their therapeutic effect, including mitochondrial transfer, RNA, and protein delivery; however, a defined and shared consensus is still missing [18]. However, interest in EV cargo is mainly fuelled by stem cell-related therapies, particularly MSCs, which are the most often examined stem cell type in clinical settings [19]. They are generally known to have low immunogenicity, regenerative properties, and immunomodulatory effects, which are primarily mediated through EVs [20].

## 3. Bioactive Molecules in Extracellular Vesicles

As outlined above, EVs transport a broad spectrum of bioactive molecules on their surface and within their lumen, including proteins, lipids, nucleic acids, and metabolites. The molecular composition of EVs reflects the identity and state of the producing cell [21]. Membrane-associated proteins, such as tetraspanins (CD9, CD63, and CD81), integrins, and adhesion molecules, are commonly involved in vesicle targeting, uptake, and intercellular communication. The intraluminal content often comprises signalling proteins, enzymes, and regulatory RNAs that mediate recipient cell function. Hence, most EV cargo is functionally linked to exosome biogenesis, cargo sorting, target cell recognition, membrane fusion, signalling pathway activation, antigen presentation, and the modulation of immune responses. In addition, some molecular components contribute to the structural integrity and rigidity of the vesicle, influencing its stability and bioavailability. These features underscore EVs’ complex and dynamic nature as mediators of intercellular communication and potential therapeutic agents [22,23].

**Figure 1:**
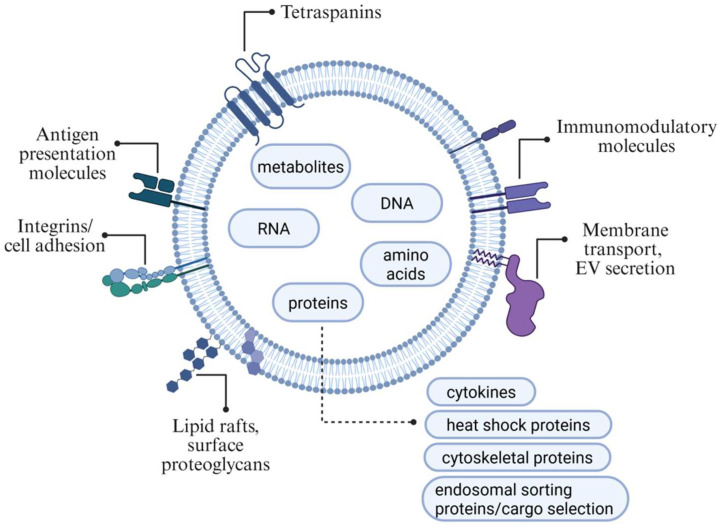
Extracellular vesicle composition: Presented are the main groups of molecules expressed on the surface of EVs. The internal cargo of the EV consists of DNA, various RNAs, amino acids, and proteins, which are more accurately presented in Table 1. Created in BioRender (2025). Abbreviations: (extracellular vesicles (EVs).

The presence of these molecules, which determine the specific functions of extracellular vesicles (EVs), varies depending on the cell type of origin and its physiological or pathological condition. Nevertheless, some molecules are commonly found across EVs released by various cell types (Figure 1) [11]. Table 1 summarises selected molecules or molecule classes generally present in EVs. Some of them are currently under investigation for their potential therapeutic applications.

Many parameters influence EV cargo, particularly when EVs are produced using in vitro cell culture. Primarily, the producing cells’ characteristics (viability, passage number, seeding, and harvest density) determine the content. At the same time, the medium composition, culture conditions (e.g., temperature, pH, and gas concentrations), and duration of conditioning also play important roles [10]. For example, Naskou et al. (2024) demonstrated that EVs produced by different MSC sources and cell culture conditions exhibit distinct phenotypes and immunomodulatory properties [27]. The biogenesis process itself (determining which EV subtype is formed) can also shape the molecular cargo carried by these vesicles, thereby theoretically influencing their distinct therapeutic roles, including their potential contributions to corneal healing [28]. However, most ocular surface regeneration studies to date utilised total EV populations due to the methodological challenges and low yields associated with potential subtype-specific isolation.

Comparing different studies, especially those involving EVs, is inherently challenging due to the variability in experimental approaches. Scientists often use different cell lines and methods for EV analysis and isolation, which can lead to discrepancies in the vesicle profiles obtained [29]. Additionally, the method chosen to analyse the cargo profile can significantly influence the results, further adding to the complexity of comparing the findings across studies [18,30].

### 3.1. Interaction of Extracellular Vesicles and Recipient Cells

The biological impact of EVs on recipient cells can result from two main modes of action: (1) receptor-mediated signalling at the cell surface without uptake, or (2) intracellular release of EV cargo following successful internalisation and endosomal escape [10].

Initial recognition and binding of EVs to the plasma membrane is often mediated by specific protein–protein interactions with membrane receptors, ligands, or contact proteins of recipient cells (such as tetraspanins, lectins, proteoglycans, and integrins) [31]. However, binding alone does not necessarily lead to internalisation, as EVs can also trigger receptor-mediated signalling without being taken up. EV uptake occurs via multiple mechanisms, including clathrin-dependent and clathrin-independent endocytosis, caveolin-mediated pathways, lipid raft-mediated internalisation, macropinocytosis, phagocytosis, and in some cases, direct fusion with the plasma membrane [10]. The choice of pathway appears to depend on both EV and recipient cell properties. Recent studies using live imaging and single-EV tracking suggest that multiple uptake routes can operate simultaneously in the same cell population, but with different efficiencies [32].

Once internalised, EVs are typically trafficked to early endosomes, where they may undergo the following: (1) recycling back to the plasma membrane, (2) degradation via late endosomes and lysosomes, or (3) endosomal escape, enabling the release of EV cargo into the cytoplasm [10,11]. Su et al. (2025), for example, recently demonstrated that milk-derived EVs in cultured cancer cells are primarily internalised through clathrin-mediated endocytosis and macropinocytosis and can evade lysosomal degradation to release their content intracellularly [33].

The relative importance of these processes remains poorly understood. Evidence indicates that EV internalisation can be inefficient in some cell types, often requiring high EV-to-cell ratios to observe measurable uptake [10]. Moreover, it is unclear whether different internalisation routes lead to distinct intracellular trafficking patterns and biological responses, or whether they converge on similar downstream effects. Another unresolved question is whether the uptake and processing of externally administered EVs in vitro mimic the endogenous mechanisms of EV-mediated communication in vivo [11]. Furthermore, while some studies report non-selective uptake of EVs by virtually any cell type [32,34], others suggest that the EV lipid composition, surface glycans, and tetraspanin profiles influence cellular tropism, thereby conferring selectivity to EV–cell interactions [35,36]. Jurgielwicz et al. also indicated that the secreted exosomes are preferentially taken up by cell types where they were originally secreted from [36]. Overall, the mechanisms underlying EV binding, uptake efficiency, intracellular trafficking, and cargo release remain incompletely elucidated.

### 3.2. Bioactive Molecules in Extracellular Vesicles Relevant to Corneal Regeneration

#### 3.2.1. MicroRNAs (miRNAs)

miRNAs are small (21–23 nucleotides) non-coding RNAs that are involved in a variety of biological processes and are the most commonly studied molecules in EVs. miRNAs regulate gene expression through several mechanisms. When miRNA binds to the target mRNA with partial complementarity, it can inhibit translation by blocking the initiation or elongation steps of protein synthesis. In cases where the miRNA has perfect or near-perfect complementarity to the target mRNA, it can induce mRNA cleavage and degradation. miRNAs can also promote the deadenylation of target mRNAs, leading to their destabilisation and subsequent degradation. miRNAs can induce chromatin modifications and DNA methylation, leading to gene silencing at the transcriptional level.

According to recently published research, miRNAs play a crucial role in regulating multiple elements of corneal disorders, including inflammation, wound healing, differentiation, and cell proliferation. However, despite advancements in pre-clinical investigations, the field of miRNA-based therapeutics remains in an early development phase, with only a handful progressing to clinical trials [37].

#### 3.2.2. Cytokines and Growth Factors

Cytokines and growth factors are small, secreted proteins that play a fundamental role in cell-to-cell communication, enabling cells to coordinate complex biological processes such as immune responses, tissue regeneration, and wound healing. These molecules exert their effects by binding to specific receptors on the surface of recipient cells, triggering intracellular signalling cascades that regulate gene expression, cell proliferation, differentiation, migration, and survival [38].

Specifically, healing of corneal epithelial defects proceeds through three stages: mitosis of limbal epithelial stem cells, migration, and their differentiation [39]. In all of these stages, multiple molecular pathways are activated, producing a series of complex and coordinated cellular processes tightly regulated by cytokines, growth factors, and proteases produced by various cells of the ocular surface such as lacrimal gland, inflammatory, corneal epithelial cells, and activated stromal fibroblasts [39].

Among the key growth factors implicated in corneal repair are connective tissue growth factor (CTGF), epidermal growth factor (EGF), hepatocyte growth factor (HGF), platelet-derived growth factor (PDGF), transforming growth factors α (TGF-α) and β (TGF-β), vascular endothelial growth factor (VEGF), and nerve growth factor (NGF). Cytokines also play a critical role, with interleukin-1 (IL-1), tumour necrosis factor-α (TNF-α), Fas ligand, interleukin-4 (IL-4), and Iinterleukin-10 (IL-10) contributing to the regulation of inflammation and tissue remodelling [39,40].

## 4. Therapeutic Effect of Extracellular Vesicle-Derived Specific Molecules on the Ocular Surface and Cornea

**Figure 2:**
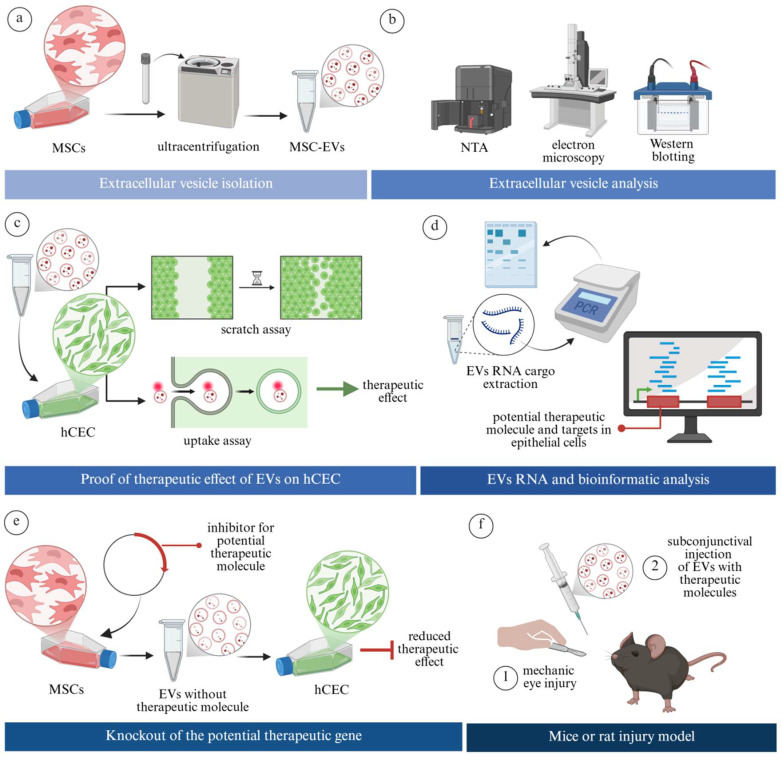
Schematic representation of an experimental study design to analyse the molecular basis of EVs’ therapeutic effect on corneal wound healing according to Liu, 2022 [41]. (**a**) MSC- or other cell-derived EVs are collected from conditioned media and isolated using methods such as ultracentrifugation. (**b**) Identity, purity, and quality of isolated EVs is confirmed with various methods, including different light scattering techniques, electron microscopy, and Western blotting. (**c**) To evaluate the potential therapeutic effect of EVs on tissue regeneration, hCECs are treated with EVs. Uptake and proliferation assays are performed to assess their effects on cellular internalisation, migration, and growth. (**d**) Total RNA from EVs and hCECs is extracted for molecular cargo identification, which could be responsible for the studied therapeutic effect. (**e**) To test whether a specific molecule is responsible for the therapeutic effect of EVs on hCECs, its gene is knocked out in MSCs. EVs are then isolated from these modified cells. Their effects on hCEC healing are compared to EVs from unmodified (wild-type) MSCs. (**f**) To evaluate the therapeutic relevance, EVs are tested in an animal rodent corneal injury in vivo model. Created in BioRender (2025). Abbreviations: mesechymal stem cells (MSC), extracellular vesicles (EVs), nanoparticle tracking analysis (NTA), and human corneal epithelial cells (hCEC).

To date, relatively few studies investigated the underlying mechanisms through which EVs exert the therapeutic effect on the ocular surface, and even fewer identified the specific bioactive molecules mediating these effects. Most published work still focuses primarily on EV administration’s overall positive therapeutic outcomes without dissecting the molecular basis of action. Figure 2 illustrates a conceptual framework for studies aimed at identifying specific pro-therapeutic components within EVs. It includes strategies such as gene knockout of potential molecules in EV-producing cells, followed by assessment of whether the therapeutic effect of the resulting EVs is diminished in a specific disease model [41].

Table 2 summarises studies that used robust research models for identifying specific molecules presumably responsible for the therapeutic effect of EVs on corneal wound healing and ocular surface stability. Except one study that isolated EV from platelets [31], all the other studies isolated EVs from MSCs of various origins [25,26,27,28,29,30,32,33,34,35,36].

### 4.1. Extracellular Vesicle Effect on Corneal Wound Healing

**Figure 3:**
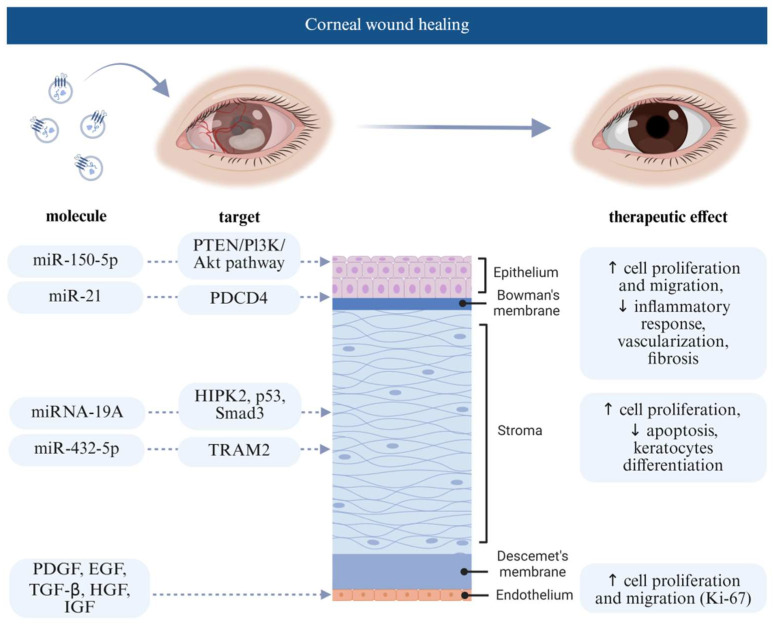
Key EV-derived molecules, their corneal cellular targets, and resultant therapeutic effects during wound healing across the epithelium, stroma, and endothelium. Created in BioRender (2025). Abbreviations: phosphatase and tensin homolog (PTEN), phosphoinositide 3-kinase (PI3K), protein kinase B (Akt), programmed cell death protein 4 (PDCD4), homeodomain-interacting protein kinase 2 (HIPK2), cellular tumour antigen p53 (p53), SMAD family member 3 (Smad3), translocation-associated membrane protein 2 (TRAM2), platelet-derived growth factor (PDGF), epidermal growth factor (EGF), transforming growth factor beta (TGF-β), hepatocyte growth factor (HGF), and insulin-like growth factor (IGF).

The majority of studies are preclinical and focus on enhancing corneal repair mechanisms (Figure 3). In 2018, Samaeekia et al. (Study No. 2) explored human corneal MSC-derived EVs for corneal epithelial wound healing. EVs accelerated corneal wound healing in human corneal epithelial cells (hCECs) by over 50% compared to the control group (*p* < 0.005). In vivo studies also demonstrated similar results, highlighting the therapeutic potential of these EVs in corneal epithelial wound healing [43]. In the same year, Shen et al. (2018) (Study No. 1) isolated EVs from adipose-derived MSCs and studied their effect on corneal stromal cells. They found out that MSC-derived EVs promoted proliferation and inhibited early apoptosis of stromal cells with downregulation of matrix metalloproteinases and upregulation of extracellular matrix-related proteins, including collagens and fibronectin in corneal stromal cells [42]. In another study Shen et al. (2020) (Study No. 3) showed that miRNA-19A from adipose MSC-derived EVs suppressed fetal bovine serum-induced differentiation of keratocytes into myofibroblasts by inhibiting HIPK2, lowering phosphorylated Smad-3, p53, collagen III, and fibronectin levels [44]. Tang et al. (Study No. 4) developed an EV-loaded thermosensitive hydrogel that promotes damaged corneal epithelium and stromal layer healing by reducing stromal extracellular matrix deposition. EVs isolated from pluripotent stem cell-derived MSCs were applied directly to the corneal wound via the hydrogel. They contained miR-432-5p, which targets and suppresses the TRAM2 gene, a vital modulator of collagen biosynthesis in corneal stromal stem cells, thus reducing corneal scar formation and accelerating the healing process [45].

In another study (Study No. 6), Liu et al. demonstrated that the EVs isolated from human umbilical cord MSC may significantly enhance corneal epithelial wound healing in vivo and corneal epithelial cell proliferation and migration in vitro. In the in vivo experiments using rats, EVs were injected subconjunctivally into the mechanically wounded eye, where corneal epithelium was removed. Based on the experimental results, they concluded that the injected human umbilical cord MSC-derived EVs, containing miR-21 promoted hCECs’ proliferation and migration, as miR-21 directly targets and downregulates the PTEN gene, thus upregulating the PI3K/Akt signalling pathway. This mechanism is closely connected to cell growth, differentiation, and apoptosis, which might lead to better corneal regeneration and wound repair [41]. Similarly, Xu et al. (Study No. 13) cultivated bone marrow MSCs in a 3D gelatin methacryloyl hydrogel to produce EVs with better corneal regenerative properties. Compared to 2D, the 3D-cultured MSCs-derived EVs were produced in better yield, exhibited strong anti-inflammatory, pro-proliferative, and tissue remodeling properties. EVs were loaded into a hydrogel and applied to a corneal wound, their therapeutic effect was linked to miR-150-5p-targeting PDCD4, promoting cell proliferation and suppressing inflammatory responses [52].

Saccu et al. (Study No. 9) further studied bone marrow MSC-derived EVs and their effect on corneal angiogenesis and inflammation in a murine model of alkali-burn-induced corneal damage. They showed that administration of these Evs created an anti-inflammatory and pro-survival environment that prevented angiogenesis in corneal tissue [4]. In another study, Zhou et al. (Study No. 10) revealed that bone marrow MSC-derived Evs promote proliferation and migration of human corneal epithelial cells via activating the p44/42 MAPK pathway in vitro. In vivo using a murine model, the treatment inhibited alkali burn-induced inflammation, fibrosis, and vascularisation in the corneal tissues, downregulating fibrosis (α-SMA) and vascularisation (CD31) in the cornea [49].

Only one study (Study No. 8) so far focused on the innermost corneal endothelial cell layer, which has very limited regenerative ability in vivo, with endothelial cells slowly decreasing in number with increasing age (31). The research group analysed platelet-derived EVs, which contained a mixture of growth factors and multiple other trophic factors, as well as proteins related to EVs with functional activities associated with cell cadherin and adherens pathways. EVs contained PDGF, EGF, TGF-β, HGF, and IGF, which are critical for corneal regeneration. EVs induced no endothelial cell toxicity and improved proliferation and migration of cultured endothelial cells in a wound-healing assay. The cultured cells expressed higher levels of Ki-67, a proliferation marker. The regeneration potential was dose-dependent. Thus, the authors concluded that platelet-derived EVs could be effective in corneal endothelial regeneration [48].

### 4.2. Extracellular Vesicle Effect on Dry Eye Disease

**Figure 4:**
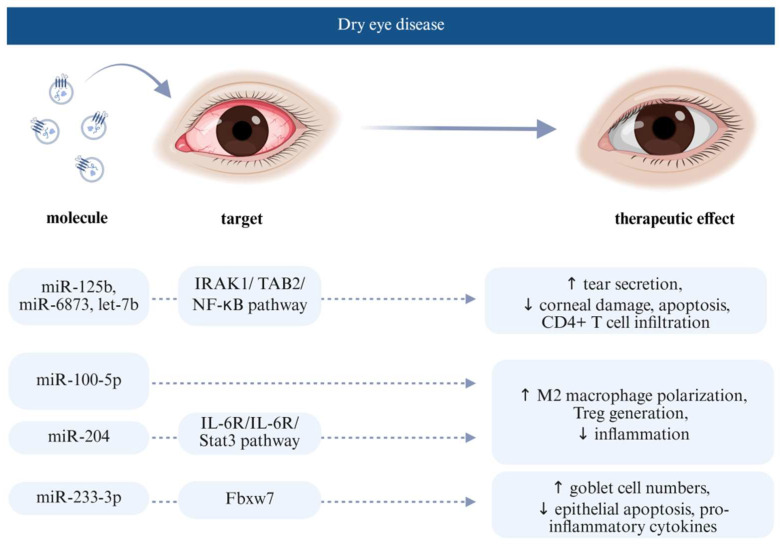
EV-derived bioactive bolecules, their cellular targets, and therapeutic effects in the pathophysiology of dry eye disease. Created in BioRender (2025). Abbreviations: interleukin-1 receptor-associated kinase 1 (IRAK1), TAK1-binding protein 2 (TAB2), nuclear factor kappa-light-chain-enhancer of activated B cells (NF-κB), interleukin 6 (IL-6), interleukin 6 receptor (IL-6R), signal transducer and activator of transcription 3 (Stat3), and F-box/WD repeat domain-containing 7 (Fbxw7).

Dry eye disease (DED) is a common chronic condition, affecting 5–50% of the population, caused by an unstable or insufficient tear film and accompanied by variable degrees of ocular surface epitheliopathy, inflammation, and neurosensory abnormalities. It leads to symptoms such as eye discomfort and blurred vision, often impairing daily functioning and quality of life. DED is typically classified into evaporative and aqueous-deficient types, though many patients experience a combination of both [53]. The current treatment methods include artificial tear replacement as well as local anti-inflammatory and immunosuppressive therapy, which are mainly limited to improve ocular surface discomfort and inflammation; thus, new treatment options are sought, such as usage of EVs (Figure 4). Wang et al. (study No. 11) evaluated the therapeutic potential of EVs derived from human umbilical cord MSCs and their miRNAs in a desiccation-induced DED mouse model. Treatment with EVs alleviated DED symptoms by increasing tear secretion, reducing corneal damage, preserving goblet cell density, and inhibiting both apoptosis and CD4+ T cell infiltration. miRNA sequencing of hucMSC-EVs identified several key miRNAs, such as miR-125b, miR-6873, and let-7b, that target upstream regulators of the NF-κB signalling pathway, including TLR4, IRAK1, TRAF6, TAB2, and NFKBIE. These targets were significantly downregulated at both the mRNA and protein levels following hucMSC-EVs treatment. hucMSC-EVs effectively inhibited inflammation on the ocular surface through multi-targeting of the IRAK1/TAB2/NF-κB signalling pathway [50]. Similarly, Li et al. (Study No. 7) injected human umbilical cord-derived MSC-EVs subconjunctivally into a rabbit dry eye model and showed that the treatment alleviated autoimmune dacryoadenitis by promoting M2 macrophage polarisation and Treg generation, possibly through miR-100-5p shuttling by EVs [47]. Moreover, Zhou et al. (Study No. 5) reported the first prospective clinical study using EVs isolated from human umbilical cord MSCs showing a promising therapeutic effect in patients with graft-versus-host disease (GVHD)-associated dry eye disease [46]. EVs were applied topically as eye drops containing miR-204 and targeting the IL-6R gene, which suppresses the activation of the IL-6R/Stat3 pathway [46]. Through this pathway, they similarly concluded that EVs reprogrammed the proinflammatory M1 macrophages toward the immunosuppressive M2 phenotype, suppressing inflammation on the ocular surface and improving epithelial healing, highlighting miR-204 as a potential therapeutic agent [46].

Wang et al. (Study No. 12) aimed to investigate the therapeutic effect of miR-223-3p derived from mouse adipose MSC-derived EVs in a dry eye model. Expression of miR-223-3p was confirmed in mouse corneal epithelial cells, mouse adipose-derived MSCs, and their EVs. In induced dry eye models, overexpression of miR-223-3p reduced fluorescein staining, thus preventing corneal epithelial damage. It also increased goblet cell numbers, reduced conjunctival epithelial apoptosis, and lowered levels of certain pro-inflammatory cytokines compared to the positive control. The authors concluded that miR-223-3p alleviates ocular surface damage and inflammation by downregulating Fbxw7, which is a direct target of miR-223-3p. Thus, miR-223-3p might be a potential new anti-inflammatory therapy for DED [51].

## 5. Extracellular Vesicles as Delivery Systems of Therapeutic Molecules

EVs can carry different cargos, including genetic substances, proteins, and small molecule drugs (curcumin, dopamine, paclitaxel, doxorubicin, and gemcitabine) [54]. Compared to their producing cells, EVs offer distinct advantages in the targeted delivery of therapeutic molecules, as their surface proteins enhance specific interactions with recipient cells, enabling more precise and potentially safer therapeutic effects. Due to their endogenous cellular origin, EVs are generally considered to be more biocompatible and less immunogenic than synthetic nanoparticles [23]. Hence, besides investigating the therapeutic effects of EV-derived therapeutic molecules, which are naturally present in the cytoplasm of producing cells, many studies use vesicles solely as delivery systems for non-endogenously produced therapeutic molecules (Table 3). Two primary methods are typically used for loading the therapeutic molecules into EVs. The first involves loading cargo after EV isolation, using electroporation or sonication, which can compromise both the EVs’ integrity and the therapeutic molecules’ structure. An alternative approach is to load cargo before EV isolation through cell sorting mechanisms [55].

EV-based drug delivery has high flexibility and compatibility because it can be delivered via intravenous, subcutaneous, intranasal, intraperitoneal injection, and oral administration. In ophthalmology, EVs can be delivered to the eye through various routes, such as topical application (e.g., eye drops), subconjunctival injection, or intravitreal injection, depending on the targeted ocular tissue and therapeutic purpose. However, even though there are many advantages of using EVs in the delivery system, there are still four major problems to be solved: (1) large-scale production, (2) isolation, (3) loading efficiency, and (4) biodistribution and uptake of EVs [23]. Some of these shortcomings could be improved by vesicle modification, including genetic modification, lipid insertion, click chemistry, metabolic labelling, affinity binding, and enzymatic ligation. Artificial vesicles can be prepared using cells and have similar structures and functions to nature EVs. Vesicles can be prepared using plasma membrane fragments, which involve the initial removal of unwanted components from a cell suspension by centrifugation, followed by mechanical or chemical disruption of the cells to isolate the cell membrane and other organelles. Compared with naturally derived EVs, vesicles prepared in this way have significantly higher yields and are more stable, making them promising candidates for drug delivery [56,57,58].

In addition to the challenges related to efficient cargo loading into EVs and their controlled delivery to target tissues, a fundamental bottleneck lies in the upstream processes of cell cultivation, EV harvesting, isolation, and characterisation [30]. Currently, most studies in this field employ slightly different culture conditions and isolation methodologies, resulting in data that are difficult to compare and reproduce—an important limitation when considering future large-scale clinical trials. Over time, we anticipate the development and optimisation of protocols, including adaptations tailored for nanoscale vesicles that will be essential for ensuring consistent production and administration of EV-derived therapeutic molecules.

**Table 3:** Summary of studies utilising EVs as delivery systems of therapeutic molecules to the ocular surface.

Therapeutic Molecule	Target	Disease	Producing Cell	Year	Reference
ocu-microRNA 24-3p (miRNA 24-3p)	rabbit corneal epithelial cells migration and corneal repair	corneal epithelial healing	human adipose-derived MSCs	2023	[59]
siRNA	/	dry eye disease	hCEC	2024	[60]
gold nanoparticles (AuNPs) reduced by ascorbic acid	/	dry eye disease	human MSC	2023	[61]
kaempferol	/	corneal neovascularisation	human platelets	2025	[62]

Abbreviations: mesenchymal stem cells (MSC)s, hCEC (human corneal epithelial cell).

## 6. Future Perspectives

Although early-stage clinical studies are evolved in several medical fields with promising results [63], to date, only one prospective clinical trial has been published using topical EVs isolated from human umbilical cord MSCs in patients with graft-versus-host disease (GVHD)-associated dry eye disease [46]. In addition, there are only a few registered clinical trials (https://clinicaltrials.gov) assessing the safety, tolerability, and efficacy of topically applied EVs for treating ocular surface or dry eye disease, using either allogeneic MSC-derived EVs (NCT05204329), pluripotent stem cell-derived MSC EVs (NCT05738629), or limbal stem cell-derived EVs (NCT06543667), with limited reports in the published literature to date. Thus, the translation of EV therapies into clinical practice still faces several safety and efficacy challenges.

One of the less explored safety concerns is the potential for unintended immunogenicity, which depends heavily on the EV cellular origin, surface protein composition, and cargo content [64]. Although the healthy eye is known as an immune-privileged site, this privilege can be lost due to ocular disease states. Thus, EVs from allogeneic or xenogeneic sources may present foreign antigens that activate innate and adaptive immune responses, leading to inflammation and accelerated EV clearance, especially after repeated administration [64]. Such immunogenic responses can limit dosing frequency and reduce long-term therapeutic benefits, emphasising the need for careful immunological profiling and engineering of EVs [64]. Furthermore, EV heterogeneity can also affect their activity, targeting, pharmacokinetics, and stability, complicating dose standardisation and manufacturing consistency [65].

## 7. Conclusions

In conclusion, while EVs hold significant promise for the treatment of various ocular surface and corneal diseases, current research remains primarily focused on their overall regenerative and anti-inflammatory potential. Only a limited number of studies have begun to explore the specific bioactive molecules responsible for these effects and the underlying mechanisms of their action. This knowledge gap is significant given that the EVs hold enormous potential for use in regenerative medicine, with the lipid bilayer of EVs protecting their molecular cargo from premature enzymatic degradation and enabling prolonged retention at the ocular surface, potentially enhancing therapeutic outcomes. Significantly, both the internal cargo and surface molecules of EVs contribute to their biological effects, which are highly dependent on the origin and physiological state of the producing cell. To fully harness the therapeutic potential of EVs, there is also a critical need to develop robust methods for their isolation and molecular characterisation. A deeper understanding of individual bioactive molecule biological roles could enable the design of EV-based therapies tailored to specific ocular conditions, potentially through genetically engineered vesicles with customised cargo.

## Figures and Tables

**Table 1:** Selected molecules (type) found in extracellular vesicles (EVs) and their functional role [23,24,25,26].

Molecule	General Function in Relation to Extracellular Vesicles
DNA CARGO	
dsDNA	Transferred to recipient cell for cell-to-cell communication (influencing gene expression), may act as an immune response trigger (DAMP).
mtDNA	Often enriched in EVs, particularly from stressed or cancerous cells.
RNA CARGO	
mRNA	Transferred to recipient cells for protein synthesis and intercellular communication.
miRNA	Gene expression regulation in recipient cells, involved in EV sorting.
tRNA	May play roles in stress responses and intercellular signalling.
rRNA	Typically minimal in EVs; may be present as fragments.
circRNA	Stable RNA molecules that can act as miRNA sponges or regulate transcription.
lncRNA	Gene regulation and chromatin remodelling in recipient cells.
sncRNA	Various small non-coding RNAs involved in gene regulation.
vault RNA	Associated with vault ribonucleoprotein particles; may influence drug resistance and signalling.
piRNA	Transposon silencing and genome stability.
Y RNA	Degradation of structured and misfolded RNAs in proximity to endosomes.
PROTEIN CARGO	
Heat shock proteins	
HSP60	Protein folding; may aid in EV cargo stability.
HSP70	Protein folding and protection; involved in EV biogenesis and cargo loading, may assist in membrane fusion.
HSP90	Protein stabilisation and signalling pathways, plays important role in cancer progression.
Integrins	Mediation of cell adhesion and signalling; influence on EV targeting (recipient cell specificity) and uptake.
Cytoskeletal proteins	
Actin	EV motility, possible role in EV biogenesis.
Myosin	Motor protein interacting with actin; may facilitate EV movement.
Four-transmembrane cross-linked proteins (tetraspanins)	
CD9	Membrane organisation and EV formation (cargo sorting).
CD63	Associated with late endosomes; involved in EV biogenesis.
CD81	Membrane fusion and signalling; role in target cell interaction.
CD82	Cell adhesion and migration.
Tspan8	Cell motility and metastasis (found in tumour-derived EVs; potential in cancer progression).
Membrane trafficking proteins	
Rab-GTPase	Vesicle trafficking and EV secretion pathways.
Annexin	Binds phospholipids; membrane organisation and EV formation (cargo selection).
Immuno-regulatory molecules	
MHC-I, MHC-II	Presents endogenous antigens; can influence immune recognition.
Other proteins	
CD106	Adhesion molecule; EV binding to endothelial cells.
ICAM-1	Adhesion molecule; facilitating EV attachment to target cells.
Beta-catenin, P120-catenin	Cell adhesion and Wnt signalling pathway (may influence recipient cell signalling).
TGF-β	Cytokine; regulation of cell growth and differentiation; may modulate immune responses.
HIF1α	Transcription factor; influences angiogenesis and metabolism in recipient cells.
Caveolin-1	Structural protein of caveolae; involved in endocytosis and signal transduction.
ALIX	Endosomal sorting and EV biogenesis; component of the ESCRT pathway in exosomes.
TSG101	ESCRT-I complex; essential for exosome formation (facilitates cargo sorting into EVs).
CD86	Co-stimulatory molecule in immune responses; can modulate T-cell activation.
Galectin 9	Modulates immune responses and apoptosis.
LIPID CARGO	
Cholesterol	Membrane fluidity and integrity; abundant in EV membranes (essential for structure and function).
Flotillin	Membrane formation and endocytosis (may influence cargo sorting).
Ceramide	Membrane curvature and EV budding (endosomal membranes).
Sphingolipids	Membrane structure and signalling; enriched in EVs.

Abbreviations: double-stranded DNA (dsDNA), damage-associated molecular pattern (DAMP), mitochondrial DNA (mtDNA)**,** messenger RNA (mRNA), microRNA (miRNA), transfer RNA (tRNA), ribosomal RNA (rRNA), circular RNA (circRNA), long non-coding RNA (lncRNA), small non-coding RNA (sncRNA), piwi-interacting RNA (piRNA), heat shock protein (HSP), cluster of differentiation (CD), Tetraspanin 8 (Tspan8), intercellular adhesion molecule 1 (ICAM-1), wingless-related integration site (Wnt), Transforming growth factor β (TGF-β), hypoxia inducible factor 1-α (HIF1α), ALG-2-interacting protein X (ALIX), endosomal sorting complexes required for transport (ESCRT), tumour susceptibility gene 101 (TSG101), Ras-associated binding GTPase (Rab-GTPase), and major histocompatibility complex (MHC).

**Table 2:** Summary of studies identifying specific molecules presumably responsible for the therapeutic effect of EVs on corneal wound healing and dry eye.

	Therapeutic Molecule	Molecular Target	Disease	Producing Cell	Model	Year	Reference
1	/	MMP	corneal stromal damage	rabbit ADSC	raCSC	2018	[42]
2	/	/	corneal wound	human cMSC	hCEC	2018	[43]
3	miR-19a	HIPK2, p53 and Smad3 pathways	corneal fibrosis	rabbit ADSC	rabbit cell culture (keratocytes and ADSC)	2020	[44]
4	miR-432-5p	TRAM2	corneal diseases	human iPSC-MSC	hCEC, rCSSC, rat	2021	[45]
5	miR-204	IL-6/IL-6R/Stat3 pathway	GVHD-associated dry eye disease	human umbilical cord MSC for clinical patients, mouse bone marrow MSC for in vitro	Fibroblast cell line, macrophage cell line mouse, clinical patients	2022	[46]
6	miR-21	PTEN/PI3K/ Akt pathway	corneal epithelial wound healing	human umbilical cord MSC	hCEC, rat	2022	[41]
7	miR-100-5p	promoting M2 polarisation and Treg generation	autoimmune dacryoadenitis	human umbilical cord MSC	rabbit	2022	[47]
8	PDGF, EGF, TGF-B, HGF, IGF	/	corneal wound healing	human platelets	hCEC	2022	[48]
9	/	anti-inflammation	corneal damage	human bone marrow MSC	hCEC, mouse	2022	[4]
10	/	p44/42 MAPK pathway	corneal wound	mouse bone marrow MSC	hCEC, mouse	2023	[49]
11	miR-125b-5b, miR-199-3p, let-7b-5p, miR-6873-5p, miR-432-5p, miR-122-5p, miR-4516	IRAK1/ TAB2/NF-κB	dry eye disease	human umbilical cord MSC	mouse	2023	[50]
12	miR-223-3p	Fbxw7	dry eye disease	mouse adipose-derived MSC	mCEC, mouse	2024	[51]
13	miR-150-5p	PDCD4	corneal diseases and injuries	human bone marrow MSC	hCEC, hCSC, rabbit	2025	[52]

Abbreviations: matrix metalloproteinases (MMP), adipose-derived stem cell (ADSC), rabbit corneal stem cell (raCSC), corneal mesenchymal stem cell (cMSC), human corneal epithelial cell (hCEC), homeodomain-interacting protein kinase 2 (HIPK2), cellular tumour antigen p53 (p53), SMAD family member 3 (Smad3), translocation-associated membrane protein 2 (TRAM2), induced pluripotent stem cell-derived mesenchymal stem cell (iPSC-MSC), rat corneal stromal stem cell (rCSSC), interleukin 6 (IL-6), interleukin 6 receptor (IL-6R), signal transducer and activator of transcription 3 (Stat3), graft-versus-host disease (GVHD), mesenchymal stem cell (MSC), phosphatase and tensin homolog (PTEN), phosphoinositide 3-kinase (PI3K), protein kinase B (Akt), macrophage 2 (M2), regulatory T cells (Treg), platelet-derived growth factor (PDGF), epidermal growth factor (EGF), transforming growth factor beta (TGF-β), hepatocyte growth factor (HGF), insulin-like growth factor (IGF), also ERK1/2, extracellular signal-regulated kinase 1/2 (p44/42), mitogen-activated protein kinase (MAPK), interleukin-1 receptor-associated kinase 1 (IRAK1), TAK1-binding protein 2 TAB2, nuclear factor kappa-light-chain-enhancer of activated B cells NF-κB, F-box/WD repeat domain-containing 7 (Fbxw7), mouse corneal epithelium cell (mCEC), programmed cell death protein 4 (PDCD4), and human corneal stem cell (hCSC).

## Data Availability

No new data were created.
